# Supplementary material for: The interactome of intact mitochondria by cross-linking mass spectrometry provides evidence for coexisting respiratory supercomplexes
Source: Mol Cell Proteomics. 2017 Dec 8;17(2):216–32. doi: 10.1074/mcp.RA117.000470 (PMC5795388; doi:10.1074/mcp.RA117.000470)
Supplement: Supplemental Data [file supp_RA117.000470_133922_0_supp_23978_fzffwf.zip › spectra_annotation/mito_DR_spectra_annotation/481-1-15-1-3-1.pdf]

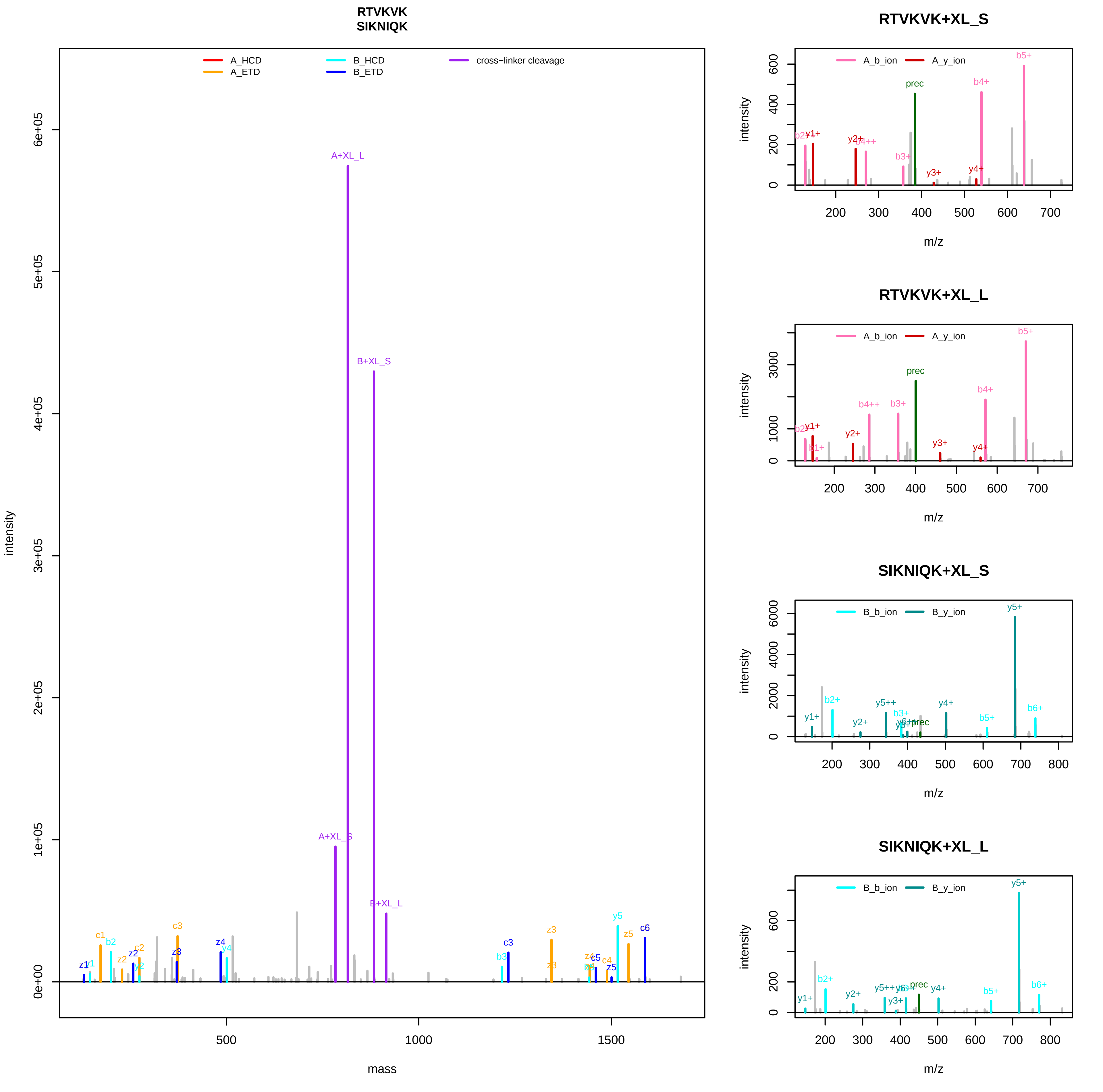
RTVKVK
SIKNIQK
A_HCD
A_ETD
B_HCD
B_ETD
cross−linker cleavage
intensity
mass
0e+00
1e+05
2e+05
3e+05
4e+05
5e+05
6e+05
500
1000
1500
A+XL_L
B+XL_S
A+XL_S
B+XL_L
z1
y1
c1
b2
z2
c2
y2
z3
c3
z4
y4
b3
c3
z3
z4
c5
c4
z5
y5
z5
c6
RTVKVK+XL_S
A_b_ion
A_y_ion
b5+
b4+
prec
y1+
y2+
b4++
b3+
y3+
y4+
m/z
RTVKVK+XL_L
A_b_ion
A_y_ion
b5+
prec
b4+
b4++
b3+
y1+
b1+
y2+
y3+
y4+
m/z
SIKNIQK+XL_S
B_b_ion
B_y_ion
y5+
b2+
y5++
y4+
b6+
y1+
b3+
y2+
b5+
prec
m/z
SIKNIQK+XL_L
B_b_ion
B_y_ion
y5+
b2+
y5++
prec
y4+
b6+
y1+
y2+
y3+
b5+
m/z
